# Early antibiotic exposure reduces progression-free survival in unresectable hepatocellular carcinoma patients treated with TACE plus TKI and ICI therapy: a propensity score-matched analysis

**DOI:** 10.1186/s12876-026-05126-4

**Published:** 2026-07-15

**Authors:** Liying Ren, Mireyi Suwanbai, Lisha Shi, Shuman Yan, Chuanzhi Deng, Weilong Xie, Li Li, Guangde Yang, Xia Wang, Xiucheng Pan

**Affiliations:** Department of Infectious Diseases and Hepatology, The Affiliated Hospital of XuZhou Medical University, XuZhou Medical University, XuZhou, JiangSu China

**Keywords:** Antibiotics, Transarterial chemoembolization, Immune checkpoint inhibitor, Tyrosine kinase inhibitor, Progression-free survival

## Abstract

**Background/objective:**

The combination of immune checkpoint inhibitors (ICIs) with transarterial chemoembolization (TACE) and tyrosine kinase inhibitors (TKIs) has emerged as a promising treatment strategy for unresectable HCC; however, the impact of concurrent antibiotic use on treatment efficacy remains controversial and poorly understood. This study aimed to evaluate the impact of early antibiotic (ATB) exposure on the long-term efficacy of TACE, TKI and ICI therapy in patients with unresectable intermediate-to-advanced HCC.

**Methods:**

We conducted a retrospective analysis of patients with hepatitis B virus (HBV)-related advanced HCC who received combination therapy with TACE, TKIs, and ICIs at the Affiliated Hospital of Xuzhou Medical University from January 1, 2019, to December 31, 2024. Patients were classified into the ATB group and non-ATB group based on whether they had received ATB therapy within 30 days prior to or 30 days after ICI initiation. The median follow-up duration was 20 months (interquartile range: 12–28 months). Follow-up assessments were performed every 3 months through outpatient reviews, re-admissions, or telephone contact.

**Results:**

After PSM, 20 matched pairs were analyzed. No significant difference in overall survival (OS) between the ATB and non-ATB groups (median OS:18.2vs24.0 months; *P* = 0.692). However, progression-free survival (PFS) was significantly shorter in the ATB group (median PFS:7.6vs12.3 months; *P* = 0.006). Multivariable analysis revealed that ATB exposure, Child-Pugh class B, and baseline HBV DNA level ≥ 2000 IU/mL were independent predictors of shorter PFS. Subgroup analyses demonstrated that the negative impact of ATB exposure on PFS was more pronounced among patients aged > 60 years, Child-Pugh class B, BCLC stage C, HBV DNA levels between 20 and 2000 IU/mL, and alpha-fetoprotein (AFP) ≥ 400 ng/mL. Incidence of adverse events (*P* = 0.266) did not significantly differ between the ATB and non-ATB groups.

**Conclusion:**

Early ATB exposure did not affect OS but was independently associated with reduced PFS in unresectable intermediate-to-advanced HCC patients receiving TACE + TKI+ICI. Prophylactic antibiotics should be used cautiously, especially in high-risk subgroups.

**Supplementary Information:**

The online version contains supplementary material available at https://doi.org/10.1186/s12876-026-05126-4.

## Introduction

 Hepatocellular carcinoma (HCC) is the sixth most common cancer globally and the third leading cause of cancer-related mortality [1]. Owing to its asymptomatic early course, the majority of patients are diagnosed at intermediate or advanced stages, precluding curative surgical resection. Multiple guidelines for hepatocellular carcinoma (HCC) diagnosis and treatment, including Chinese guidelines, generally recommend systemic therapy for patients with Barcelona Clinic Liver Cancer (BCLC) stage B or C disease who progress despite intra-arterial treatment [2]. Current standard-of-care options for advanced or unresectable HCC include locoregional therapies such as transarterial chemoembolization (TACE), molecularly targeted agents including tyrosine kinase inhibitors (TKIs), and immune checkpoint inhibitors (ICIs), notably anti–PD-1 monoclonal antibodies. Emerging evidence from clinical trials indicates that ICI-based combination regimens confer substantial antitumor activity and prolong overall survival (OS) [3–5].

The majority of patients with HCC have underlying cirrhosis. Those with decompensated disease are especially susceptible to bacterial and fungal infections because of compromised immunity, resulting in frequent hospitalizations and increased mortality. Consequently, early antibiotic therapy is commonly initiated either therapeutically or prophylactically, even in the absence of confirmed infection, in an effort to prevent hospital-acquired infections and sepsis [6, 7].

The impact of antibiotic exposure on targeted therapy and immunotherapy in HCC remains contentious. Pre-clinical and clinical data suggest that antibiotics may attenuate immunotherapeutic efficacy by perturbing intestinal microbiota and disrupting the gut–liver axis [8–10]. Conversely, others contend that the baseline dysbiosis characteristic of chronic liver disease could render additional antibiotic-induced microbiota alterations clinically irrelevant [11].

Accordingly, we conducted this study to delineate the effect of early antibiotic exposure on clinical outcomes in patients with HBV-related advanced HCC treated with the TACE–TKI–ICI triad. By evaluating both progression-free survival (PFS) and overall survival (OS), we sought to generate evidence supporting the hypothesis that antibiotic-induced microbiota modulation influences immunotherapeutic efficacy and to inform evidence-based antibiotic stewardship in this setting.

## Materials and methods

### Patients

We consecutively enrolled patients with advanced HCC who received TACE combined with TKIs and ICIs at the Affiliated Hospital of Xuzhou Medical University from 1 January 2019 to 31 December 2024. Immune checkpoint inhibitors (ICIs) included camrelizumab (Hengrui Medicine, Jiangsu, China), sintilimab (Innovent Biologics, China), and tislelizumab (BeiGene, Beijing, China), administered at a fixed dose of 200 mg every 3 weeks (Q3W). The TACE protocol and choice of embolic agent were tailored to tumour burden and residual hepatic function. TKIs (sorafenib, lenvatinib, apatinib or anlotinib) were selected at the treating physician’s discretion. Inclusion criteria were: (1) age ≥ 18 years; (2) HCC was diagnosed according to the 2022 American Association for the Study of Liver Diseases (AASLD) Clinical Practice Guidance; (3) completion of ≥ 3 ICI cycles; (4) positive serum HBsAg(+), receiving antiviral therapy or documented previous HBV infection; (5) BCLC stage B or C; (6) Child-Pugh class A or B (score ≤ 7). Patients with Child-Pugh class C were excluded due to poor liver reserve and high treatment-related mortality risk. Patients were excluded if they: (1) survived < 3 months from treatment initiation (*n* = 17; see Supplementary Table S1 for baseline characteristics and outcomes of these patients); (2) had concurrent malignancies; (3) lacked complete baseline or follow-up data; or (4) presented with severe bleeding or active infection at baseline.

### Study design and assessments

Definition of antibiotic exposure window: Individuals were assigned to the antibiotics (ATB) cohort if they received any antibiotic within 30 days before or after the first ICI infusion; otherwise they were allocated to the non-ATB cohort. Systemic antibiotic use within 30 days before or after anticancer therapy initiation was recorded. This 30-day window was selected based on prior studies by Pinato et al. [12] and Elkrief et al. [13], which identified this period as critical for antibiotic-induced gut dysbiosis and immunotherapy efficacy. Antibiotics comprised carbapenems, cefoperazone-sulbactam, cefatriaxone, piperacillin, moxifloxacin and levofloxacin. Overall survival was calculated from the date of first ICI dosing to death from any cause, last known follow-up, or database lock. Adverse events were graded with CTCAE version 5.0.

### Tumor response evaluation

We retrospectively assessed treatment responses according to the modified Response Evaluation Criteria in Solid Tumors (mRECIST) for HCC. Contrast-enhanced CT or MRI was performed every 6–8 weeks during the first 6 months and every 8–12 weeks thereafter. Only viable tumor showing arterial phase hyperenhancement was measured; necrotic or non-enhancing areas were excluded. Best overall response (BOR) was defined as the best response recorded from the start of treatment until disease progression or initiation of new anticancer therapy. Response categories included complete response (CR), partial response (PR), stable disease (SD), and progressive disease (PD). Objective response rate (ORR) was defined as CR + PR, and disease control rate (DCR) as CR + PR + SD. Complete response required complete disappearance of arterial enhancement in all target lesions; in this cohort of predominantly advanced-stage HCC with portal vein tumor thrombosis (20.1%) and elevated baseline AFP (41.96% ≥400 ng/mL), no patient achieved CR, which is consistent with published real-world data for similar populations. Detailed mRECIST response data are provided in Supplementary Table S2.

### Treatment protocol and sequencing

All patients received TACE combined with ICI and TKI therapy in a concurrent (synchronous) manner. The median number of TACE sessions was 4 (interquartile range: 2–5; range: 1–11) per patient in the full cohort. TACE sessions were performed every 6–8 weeks at the treating physician’s discretion based on tumor response, residual hepatic function, and patient performance status. ICI was initiated within 2 weeks after the first TACE session and administered at a fixed dose of 200 mg every 3 weeks. ICI therapy was not routinely withheld for subsequent TACE sessions. It was continued on schedule unless the patient experienced intolerable adverse events or grade ≥ 3 treatment-related toxicities requiring hospitalization, with re-initiation upon recovery to grade ≤ 1. TKI administration was maintained continuously throughout the treatment course. The three anti-PD-1 antibodies used were camrelizumab (Hengrui Medicine, Jiangsu, China), sintilimab (Innovent Biologics, China), and tislelizumab (BeiGene, Beijing, China), administered at a fixed dose of 200 mg every 3 weeks. The distribution of ICI types was comparable between the ATB and non-ATB groups before matching (*P* = 0.519) and identical after propensity-score matching (*P* = 1.000) (Supplementary Table S3). Due to the limited matched sample size (*n* = 40) and the balanced distribution of ICI types, subgroup analyses by individual ICI agent were not performed.

### Data collection

Clinical information was retrieved from the hospital’s electronic medical record system and included demographics, HBV serostatus, liver biochemistry, hematology, serum alpha-fetoprotein, coagulation tests, radiology reports, and details of ICI and antibiotic therapies. Follow-up data were refreshed every 3 months through outpatient reviews, re-admissions or telephone contact.Study duration and follow-up: The overall study duration was from January 1, 2019, to December 31, 2024. The median follow-up duration was 20 months (range: 3–58 months), with the last follow-up date being December 31, 2024. Follow-up data were refreshed every 3 months through outpatient reviews, re-admissions or telephone contact.

### Statistical analysis

Statistical analyses were performed with SPSS 26.0. Continuous variables with a normal distribution are expressed as mean ± SD and were compared using the Student t test; skewed data are presented as median (25th–75th percentile) and were compared with the Mann-Whitney U test. Categorical variables were summarised as frequencies (percentages) and compared by the χ² test. Survival curves were constructed with the Kaplan-Meier method and compared by the log-rank test. One-to-one propensity-score matching was employed to mitigate baseline confounding. Post-baseline treatment variables (number of TACE sessions, cumulative ICI cycles [first 12 cycles], and TKI duration) were excluded from the propensity-score matching model because they are time-varying variables that may be affected by treatment response, disease progression, and antibiotic exposure, potentially introducing selection bias and overadjustment if included. These variables were compared between matched groups post hoc to assess residual confounding. Baseline characteristics and outcomes of patients excluded due to early mortality (< 3 months) are provided in Supplementary Table S1. This retrospective study did not include a formal a priori sample size calculation; the sample size was determined by the number of eligible patients receiving TACE + TKI+ICI at our center between January 2019 and December 2024. Post-hoc power analysis indicated that the propensity-score matched cohort (*n* = 40), with 38 progression or death events over a median follow-up of 20 months and an observed PFS hazard ratio of 2.76 (95% CI 1.31–5.83, *P* = 0.006), provided approximately 89% power to detect a hazard ratio of ≥ 2.5 at a two-sided α of 0.05 (Schoenfeld formula). All tests were two-sided, and *P* < 0.05 was considered statistically significant.

## Results

### Baseline characteristics of patients

**Fig. 1:**
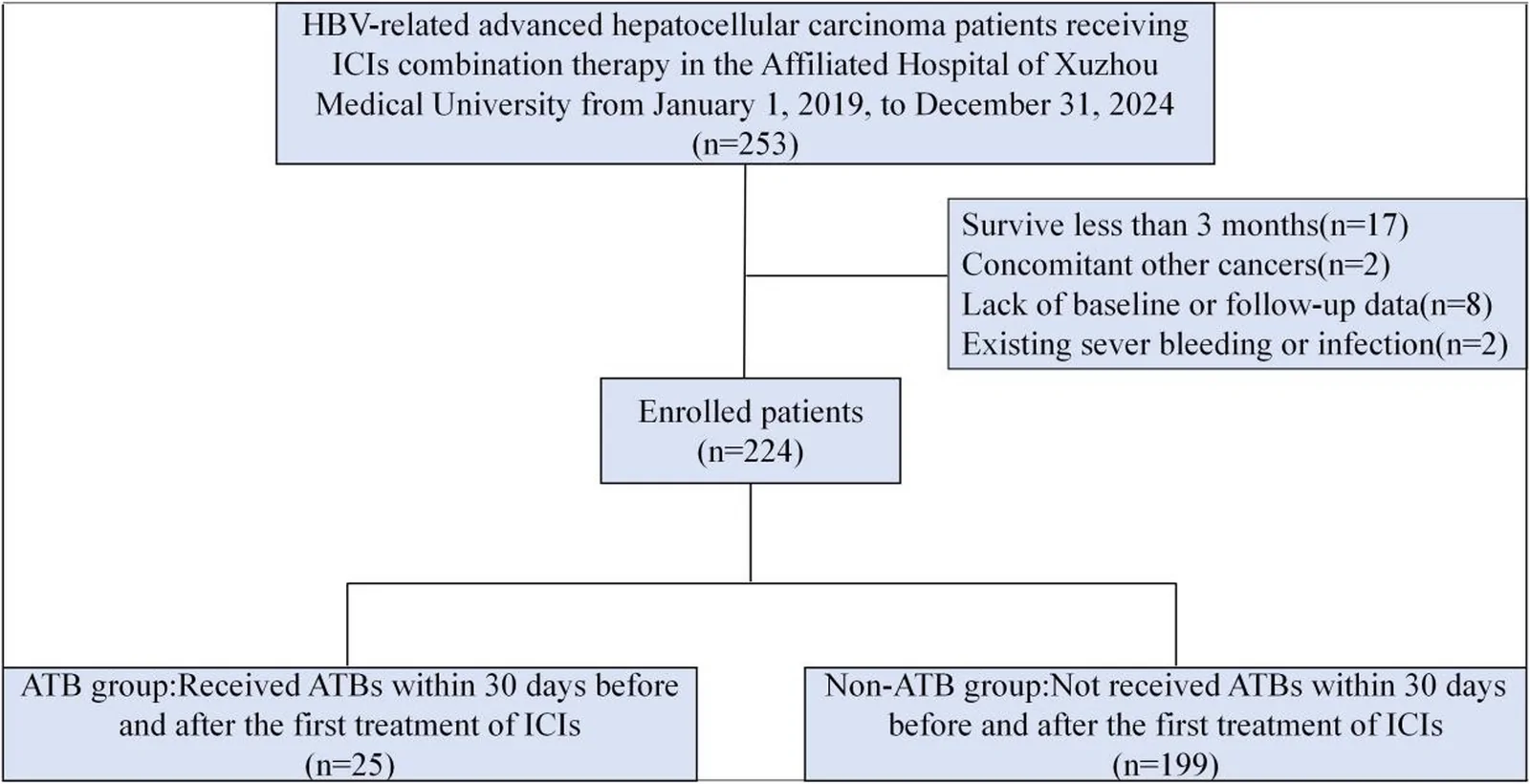
The patients flowchart. Abbreviations: ICIs, immune checkpoint inhibitors; ATB, Antibiotic; HBV, Hepatitis B virus

A total of 253 patients with HBV-related advanced HCC receiving TACE in combination with TKIs plus ICIs therapy were screened. Based on the inclusion and exclusion criteria, 29 patients were excluded: 17 survived < 3 months, 2 had concurrent malignancies, 2 presented with severe bleeding or active infection, and 8 lacked complete baseline data, and 224 patients were finally enrolled (Fig. 1). Baseline characteristics and outcomes of the 17 patients with early mortality are detailed in Supplementary Table S1. In the full cohort, twenty-five individuals received antibiotics within 30 days of the first ICI treatment (ATB cohort; Table 1), the median number of TACE sessions was 4 (IQR: 2–5). Before matching, the ATB group had a slightly lower median TACE count than the non-ATB group [3 (2–4) vs. 4 (3–6); *P* = 0.095]. After propensity-score matching, TACE exposure was well balanced between the two groups [3 (2–5) vs. 3.5 (2–4); *P* = 0.880]. The median interval between TACE sessions was 7.0 weeks (IQR: 6.0–8.0 weeks). Portal vein tumor thrombosis was present in 45 patients (20.1%) in the full cohort and was equally distributed after matching (20.0% vs. 25.0%; *P* = 1.000). Pre-matching comparisons revealed significant differences between ATB and Non-ATB group in white-cell count, sex ratio and treatment regimen. To minimise bias, 1:1 propensity-score matching was performed incorporating age, sex, treatment modality, alcohol use, Child-Pugh class, BCLC stage, HBV DNA level, antiviral status, ALT, AST, GGT, ALP, TBIL, ALB, PT, WBC, PLT and AFP. Twenty well-matched pairs were created; after matching, all baseline variables were balanced (Table 2).

**Table 1:** The use of ATB before and after PSM

Variables	Before PSM	After PSM
	Total (*n* = 25)	Total (*n* = 20)
Types, *n*(%)		
Fluoroquinolones	6 (24.00)	5 (25.00)
β-lactam	19 (76.00)	15(75.00)
Indications, *n*(%)		
Abdominal infection	7 (28.00)	5(25.00)
Bloodstream infection	6 (24.00)	5(25.00)
Other infection	4 (16.00)	3 (15.00)
Prophylactic use	2 (8.00)	1(5.00)
pulmonary infection	6 (24.00)	6 (30.00)
Duration of Antibiotic Therapy, *n*(%)		
> 7days	6 (24.00)	4 (20.00)
≤ 7days	19 (76.00)	16 (80.00)

*Abbreviations*: *ATB* antibiotics, *PSM* propensity score matching, *n* number

Types include fluoroquinolones (levofloxacin, moxifloxacin) and β-lactams (carbapenems, cefoperazone-sulbactam, ceftriaxone, piperacillin). Indications were determined by the treating physician based on clinical presentation and microbiological results. Duration ≤ 7 days vs. > 7 days was categorized based on the total antibiotic exposure within the 30-day window

**Table 2:** Baseline Characteristics of Patients Before and After Propensity Score Matching

Variables	Before PSM				After PSM			
	Total(*n* = 224)	No(*n* = 199)	Yes (*n* = 25)	*P*	Total(*n* = 40)	No(*n* = 20)	Yes (*n* = 20)	*P*
Age, years	57.00 (51.00, 65.00)	57.00 (51.00, 65.00)	58.00 (49.00, 67.00)	0.999	57.50 (50.75, 65.25)	58.00 (53.50, 64.00)	57.00 (49.75, 67.00)	0.839
<60	132 (58.93)	119 (59.80)	13 (52.00)	0.455	22 (55.00)	11 (55.00)	11 (55.00)	1
>60	92 (41.07)	80 (40.20)	12 (48.00)		18 (45.00)	9 (45.00)	9 (45.00)	
Gender, *n*(%)				0.017				1
Female	26 (11.61)	19 (9.55)	7 (28.00)		7 (17.50)	3 (15.00)	4 (20.00)	
Male	198 (88.39)	180 (90.45)	18 (72.00)		33 (82.50)	17 (85.00)	16 (80.00)	
Alcohol, *n*(%)				0.09				1
No	165 (73.99)	143 (72.22)	22 (88.00)		35 (87.50)	17 (85.00)	18 (90.00)	
Yes	58 (26.01)	55 (27.78)	3 (12.00)		5 (12.50)	3 (15.00)	2 (10.00)	
ChildPugh, *n*(%)				0.397				0.752
A	134 (59.82)	121 (60.80)	13 (52.00)		19 (47.50)	10 (50.00)	9 (45.00)	
B	90 (40.18)	78 (39.20)	12 (48.00)		21 (52.50)	10 (50.00)	11 (55.00)	
BCLC, *n*(%)				0.122				0.723
B	95 (42.41)	88 (44.22)	7 (28.00)		11 (27.50)	6 (30.00)	5 (25.00)	
C	129 (57.59)	111 (55.78)	18 (72.00)		29 (72.50)	14 (70.00)	15 (75.00)	
AntiHBV, *n*(%)				0.407				1
No	70 (31.25)	64 (32.16)	6 (24.00)		9 (22.50)	4 (20.00)	5 (25.00)	
Yes	154 (68.75)	135 (67.84)	19 (76.00)		31 (77.50)	16 (80.00)	15 (75.00)	
HBVDNA, *n*(%)				0.572				0.751
<20 IU/mL	84 (37.50)	77 (38.69)	7 (28.00)		12 (30.00)	6 (30.00)	6 (30.00)	
≥ 2000 IU/mL	57 (25.45)	50 (25.13)	7 (28.00)		14 (35.00)	8 (40.00)	6 (30.00)	
20-2000 IU/mL	83 (37.05)	72 (36.18)	11 (44.00)		14 (35.00)	6 (30.00)	8 (40.00)	
AFP, n(%)				0.521				0.744
<400 ng/mL	130 (58.04)	114 (57.29)	16 (64.00)		25 (62.50)	12 (60.00)	13 (65.00)	
≥ 400 ng/mL	94 (41.96)	85 (42.71)	9 (36.00)		15 (37.50)	8 (40.00)	7 (35.00)	
ALT, U/L	37.00 (24.00, 54.25)	37.00 (24.00, 55.50)	34.00 (24.00, 50.00)	0.644	33.00 (21.75, 53.75)	27.50 (21.75, 53.75)	38.00 (23.25, 52.25)	0.457
AST, U/L	41.00 (29.00, 62.25)	41.00 (29.00, 63.00)	45.00 (33.00, 56.00)	0.843	45.00 (28.75, 58.00)	45.00 (27.25, 65.25)	42.50 (32.75, 53.00)	0.871
GGT, U/L	80.50 (45.75, 149.00)	81.00 (46.00, 151.00)	79.00 (42.00, 125.00)	0.547	76.00 (33.50, 128.75)	75.00 (27.25, 136.75)	83.50 (40.50, 115.75)	0.665
ALP, U/L	108.50 (80.75, 146.50)	109.00 (79.00, 145.00)	104.00 (85.00, 153.00)	0.576	119.00 (81.00, 162.25)	125.50 (78.50, 173.50)	113.00 (83.25, 154.25)	0.871
Tbil, umol/L	17.20 (12.70, 24.70)	17.10 (12.78, 24.72)	18.00 (12.70, 22.80)	0.933	16.75 (12.55, 25.17)	15.00 (11.90, 22.45)	21.55 (12.85, 28.95)	0.245
Albumin, g/L	38.95 ± 5.37	39.16 ± 5.13	37.34 ± 6.90	0.214	37.75 ± 5.29	37.74 ± 4.22	37.76 ± 6.30	0.214
PT, sec	12.20 (11.50, 13.00)	12.10 (11.50, 12.90)	12.70 (11.80, 14.70)	0.025	12.90 (11.50, 14.62)	12.45 (11.50, 13.85)	13.45 (11.68, 14.93)	0.465
WBC, 109/L	4.85 (3.68, 6.60)	4.80 (3.60, 6.35)	6.00 (4.60, 9.30)	0.006	5.45 (4.55, 8.12)	6.05 (4.75, 8.38)	5.00 (4.18, 7.20)	0.317
NE, 109/L	2.98 (2.17, 4.45)	2.96 (2.15, 4.39)	3.81 (2.38, 7.51)	0.045	3.32 (2.35, 5.98)	3.55 (2.61, 6.03)	2.77 (2.27, 5.82)	0.646
LYM, 109/L	1.00 (0.70, 1.40)	1.00 (0.70, 1.40)	1.30 (0.80, 1.60)	0.106	1.20 (0.80, 1.50)	1.10 (0.78, 1.42)	1.30 (0.88, 1.65)	0.464
PLT, 109/L	129.50 (82.75, 186.75)	125.00 (81.00, 184.50)	139.00 (123.00, 204.00)	0.106	150.00 (123.75, 225.75)	197.50 (130.00, 259.00)	135.50 (120.50, 176.00)	0.081
NLR, n(%)				0.856				0.327
<2.5	77 (34.38)	68 (34.17)	9 (36.00)		15 (37.50)	6 (30.00)	9 (45.00)	
≥ 2.5	147 (65.62)	131 (65.83)	16 (64.00)		25 (62.50)	14 (70.00)	11 (55.00)	
TACE, sessions	4.00 (2.00, 5.00)	4.00 (3.00, 6.00)	3.00 (2.00, 4.00)	0.095	3.00 (2.00, 4.25)	3.00 (2.00, 5.00)	3.50 (2.00, 4.00)	0.880
PVT, n(%)				0.605				1.00
no	179 (79.91)	160 (80.40)	19 (76.00)		31 (77.50)	16 (80.00)	15 (75.00)	
yes	45 (20.09)	39 (19.60)	6 (24.00)		9 (22.50)	4 (20.00)	5 (25.00)	
PCT, ng/mL	0.14 (0.09, 0.20)	0.13 (0.09, 0.20)	0.15 (0.12, 0.20)	0.083	0.16 (0.13, 0.24)	0.18 (0.14, 0.24)	0.15 (0.12, 0.17)	0.147

*Abbreviations*: *PSM* propensity score matching, *ATB* antibiotics, *M* median, *Q*₁ first quartile, *Q*₃ third quartile, *ALT* alanine aminotransferase, *AST* aspartate aminotransferase, *GGT* gamma-glutamyl transferase, *ALP* alkaline phosphatase, *TBIL* total bilirubin, *ALB* albumin, *PT* prothrombin time, *WBC* white blood cell, *NE* neutrophil, *LYM* lymphocyte, *PLT* platelet, *NLR* neutrophil-to-lymphocyte ratio, *AFP* alpha-fetoprotein, *HBV* hepatitis B virus, *BCLC* Barcelona Clinic Liver Cancer, *TACE* transarterial chemoembolization, *PVT* portal vein tumor thrombosis, *PCT* procalcitonin

### Treatment exposure and study duration

In the full cohort, the median number of TACE sessions was 4 (IQR: 2–5; range: 1–11) per patient. TACE sessions were performed at a median interval of 7.0 weeks (IQR: 6.0–8.0 weeks). Before propensity-score matching, the ATB group had a numerically lower median TACE count than the non-ATB group [3 (2–4) vs. 4 (3–6); *P* = 0.095]. After matching, TACE exposure was well balanced between the two groups [3 (2–5) vs. 3.5 (2–4); *P* = 0.880]. The study ended on December 31, 2024, with a median follow-up duration of 20 months (range: 3–58 months). By the end of the study, 143 deaths had occurred (63.8% of the full cohort), and disease progression had been observed in 178 patients (79.5%). The distribution of ICI types was well balanced after propensity-score matching: camrelizumab (85.0% vs. 80.0%), sintilimab (10.0% vs. 15.0%), and tislelizumab (5.0% vs. 5.0%) in the ATB and non-ATB groups, respectively (*P* = 1.000; Supplementary Table S3).After propensity-score matching, the median number of TACE sessions was comparable between groups [3 (2–5) vs. 3.5 (2–4); *P* = 0.880]. Among patients with ICI tracking within the first 12 cycles, the median number of ICI cycles was 12 in both groups; however, the interquartile range suggested a broader distribution in the ATB group [12 (4–12) vs. 12 (11.25–12); *P* = 0.297], likely reflecting earlier treatment discontinuation due to disease progression or infection-related complications in the ATB cohort. TKI administration was maintained continuously throughout the treatment course in both groups. These findings indicate that any differential ICI exposure after matching was a consequence of antibiotic-related treatment failure rather than a pre-existing confounder.

### Antibiotic administration shortened the PFS of HCC patients

**Fig. 2:**
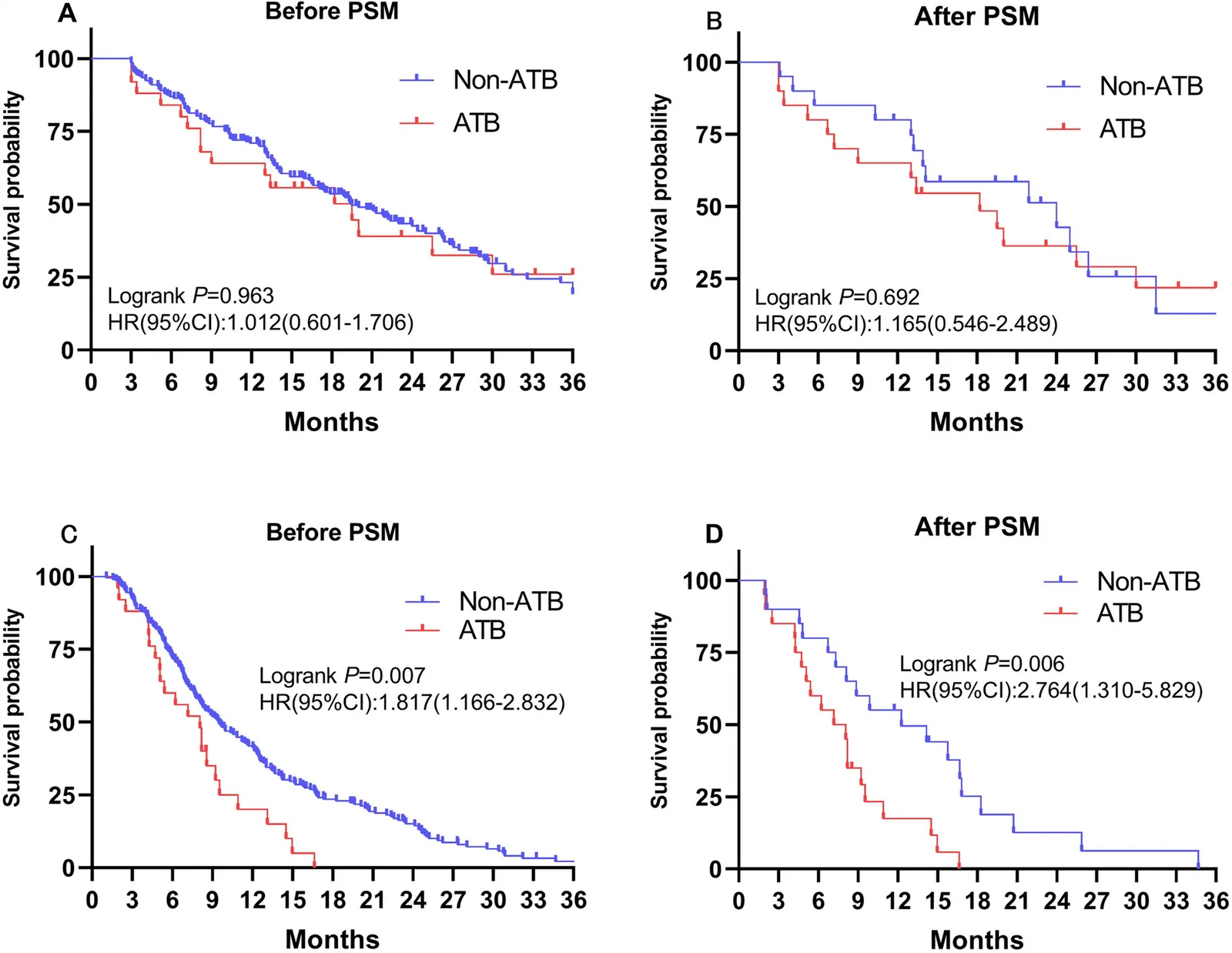
Kaplan–Meier curve for OS and PFS in patients before and after PSM (**A**) OS for patients with and without ATBs before PSM. (**B**) OS for patients with and without ATBs after PSM. (**C**) PFS for patients for with and without ATBs before PSM. (**D**) PFS for patients with and without ATBs after PSM. Abbreviations: ATB, Antibiotic, PSM, Propensity score matching ; OS, overall survival ; PFS, progression-free survival

In the full cohort, median overall survival was 19.5 months and 143 deaths were recorded. No difference in OS was seen between the ATB and non-ATB groups before matching (median 19.5 vs. 19.5 months; *P* = 0.963; HR = 1.012, 95% CI 0.601–1.706) (Fig. 2A). After propensity-score matching (median follow-up 20 months), OS remained similar between the two groups.(18.2 vs. 24.0 months; *P* = 0.692; HR = 1.176, 95% CI 0.546–2.489) (Fig. 2). In contrast, progression-free survival was significantly shorter in the ATB cohort both before matching (median 8.1 vs. 9.5 months; *P* = 0.007; HR = 1.817, 95% CI 1.166–2.832) and after matching (7.6 vs. 12.3 months; *P* = 0.006; HR = 2.764, 95% CI 1.310–5.829) (Fig. 2C, D).

### Sensitivity analysis by infection severity

To evaluate whether the observed PFS difference was driven by infection severity rather than antibiotic exposure per se, we performed a stratified analysis by baseline procalcitonin (PCT) levels. PCT is a highly specific biomarker of bacterial infection severity; levels < 0.25 ng/mL are strongly associated with a decreased likelihood of systemic bacterial infection and reduced disease severity, whereas levels ≥ 0.25 ng/mL suggest more substantial infectious burden [14, 15]. Among the 85% of matched patients with PCT < 0.25 ng/mL, indicating minimal systemic infectious burden, ATB exposure remained significantly associated with shorter PFS (HR = 2.85, 95% CI 1.24–6.56; *P* = 0.014), with an effect magnitude nearly identical to the overall cohort (HR = 2.76). The PCT ≥ 0.25 ng/mL subgroup (*n* = 6) was too small for reliable inference. There was no significant interaction between PCT level and ATB exposure (P for interaction = 0.647), though this test was underpowered. These findings suggest that the detrimental effect of antibiotics on PFS is not fully explained by differential infection severity (Supplementary Table S4).

### Tumor response according to mRECIST

According to mRECIST criteria, the best overall response in the full cohort was PR in 86 patients (38.39%), SD in 107 (47.77%), and PD in 31 (13.84%); no patient achieved CR. ORR was 38.39% and DCR was 86.16%. Before matching, the ATB group had a numerically lower ORR than the non-ATB group [32.00% vs. 39.20%] and lower DCR [76.00% vs. 87.44%]. After propensity-score matching, this trend persisted: ORR was 25.00% (5/20) in the ATB group versus 50.00% (10/20) in the non-ATB group, and DCR was 80.00% versus 90.00%; however, neither difference reached statistical significance (*P* = 0.327). Despite the lack of statistical significance—likely attributable to the modest matched sample size (*n* = 40)—the consistent direction of lower response rates in the ATB group supports the significantly shorter PFS observed in our primary analysis. Detailed response distributions are provided in Supplementary Table S2.

### Independent predictors of PFS

Cox regression analyses were performed before and after matching. In the pre-matching set, ATB exposure (HR = 1.82; 95% CI 1.17–2.83; *P* = 0.008), Child-Pugh class B (HR = 1.66; 95% CI 1.26–2.20; *P* < 0.001), HBV DNA ≥ 2000 IU/mL (HR = 1.53; 95% CI 1.07–2.19; *P* = 0.020), AFP ≥ 400 ng/mL (HR = 1.64; *P* = 0.001) and BCLC stage C (HR = 1.42; *P* = 0.014) were independently associated with shorter PFS, whereas age, sex and alcohol use were not (Table 3). After matching, the adverse effect of ATB exposure was accentuated (HR = 2.76; 95% CI 1.31–5.83; *P* = 0.008), and Child-Pugh class B (HR = 2.06; 95% CI 1.03–4.08; *P* = 0.040) and HBV DNA ≥ 2000 IU/mL (HR = 3.20; 95% CI 1.30–7.89; *P* = 0.011) remained independent predictors. However, BCLC stage C was not statistically significant (HR = 1.61; *P* = 0.237) (Table 4).

**Table 3:** Cox regression univariate and multivariate analysis of PFS before PSM

Variables	Univariate					Multivariate				
	β	S.E	Z	*P*	HR (95%CI)	β	S.E	Z	*P*	HR (95%CI)
ATB										
No					1.00(Reference)					1.00 (Reference)
Yes	0.6	0.23	2.64	0.008	1.82 (1.17 ~ 2.83)	0.69	0.24	2.93	0.003	2.00 (1.26 ~ 3.17)
AGE										
<60					1.00 (Reference)					1.00 (Reference)
>60	-0.11	0.14	-0.74	0.46	0.90 (0.68 ~ 1.19)	0.06	0.15	0.39	0.699	1.06 (0.79 ~ 1.43)
Gender										
Female					1.00 (Reference)					1.00 (Reference)
Male	0.02	0.23	0.09	0.927	1.02 (0.65 ~ 1.61)	0.31	0.24	1.29	0.198	1.37 (0.85 ~ 2.21)
Alcohol										
No					1.00 (Reference)					1.00 (Reference)
Yes	-0.22	0.16	-1.35	0.178	0.80 (0.59 ~ 1.10)	-0.21	0.17	-1.22	0.223	0.81 (0.58 ~ 1.13)
ChildPugh										
A					1.00 (Reference)					1.00 (Reference)
B	0.51	0.14	3.58	< 0.001	1.66 (1.26 ~ 2.20)	0.59	0.17	3.47	< 0.001	1.81 (1.30 ~ 2.53)
BCLC										
B					1.00 (Reference)					1.00 (Reference)
C	0.35	0.14	2.44	0.014	1.42 (1.07 ~ 1.88)	0	0.17	0	0.997	1.00 (0.72 ~ 1.39)
HBVDNA, IU/ml										
<20					1.00 (Reference)					1.00 (Reference)
≥ 2000	0.43	0.18	2.33	0.02	1.53 (1.07 ~ 2.19)	0.53	0.2	2.63	0.008	1.70 (1.15 ~ 2.52)
20-2000	0.18	0.16	1.08	0.279	1.19 (0.87 ~ 1.64)	0.35	0.17	2.01	0.044	1.42 (1.01 ~ 2.00)
AFP, ng/ml										
<400					1.00 (Reference)					1.00 (Reference)
≥ 400	0.49	0.14	3.42	< 0.001	1.64 (1.23 ~ 2.18)	0.46	0.15	3.02	0.003	1.58 (1.17 ~ 2.13)

*Abbreviations*: *PFS* progression-free survival, *PSM* propensity score matching, β regression coefficient, *S.E* standard error, *Z* Wald statistic, *HR* hazard ratio, *CI* confidence interval, *ATB* antibiotics, *HBV* hepatitis B virus, *BCLC* Barcelona Clinic Liver Cancer, *AFP* alpha-fetoprotein

**Table 4:** Cox regression univariate and multivariate analysis of PFS after PSM

Variables	Univariate					Multivariate				
	β	S.E	Z	*P*	HR (95%CI)	β	S.E	Z	*P*	HR (95%CI)
ATB										
No					1.00 (Reference)					1.00 (Reference)
Yes	1.02	0.38	2.67	0.008	2.76 (1.31 ~ 5.83)	1.42	0.41	3.46	< 0.001	4.12 (1.85 ~ 9.21)
ChildPugh										
A					1.00 (Reference)					1.00 (Reference)
B	0.72	0.35	2.06	0.04	2.06 (1.03 ~ 4.08)	1.1	0.42	2.6	0.009	3.00 (1.31 ~ 6.89)
BCLC										
B					1.00 (Reference)					1.00 (Reference)
C	0.48	0.4	1.18	0.237	1.61 (0.73 ~ 3.56)	-0.39	0.5	-0.77	0.442	0.68 (0.25 ~ 1.82)
HBVDNA, IU/ml										
<20					1.00 (Reference)					1.00 (Reference)
≥ 2000	1.16	0.46	2.53	0.011	3.20 (1.30 ~ 7.89)	1.47	0.5	2.93	0.003	4.35 (1.63 ~ 11.62)
20-2000	0.75	0.44	1.7	0.089	2.12 (0.89 ~ 5.03)	0.89	0.49	1.81	0.07	2.43 (0.93 ~ 6.38)

*Abbreviations*: *PFS* progression-free survival, *PSM* propensity score matching, *β* regression coefficient, *S.E* standard error, *Z* Wald statistic, *HR* hazard ratio, *CI* confidence interval, *ATB* antibiotics, *HBV* hepatitis B virus, *BCLC* Barcelona Clinic Liver Cancer

### Subgroup analyses for OS and PFS

**Fig. 3:**
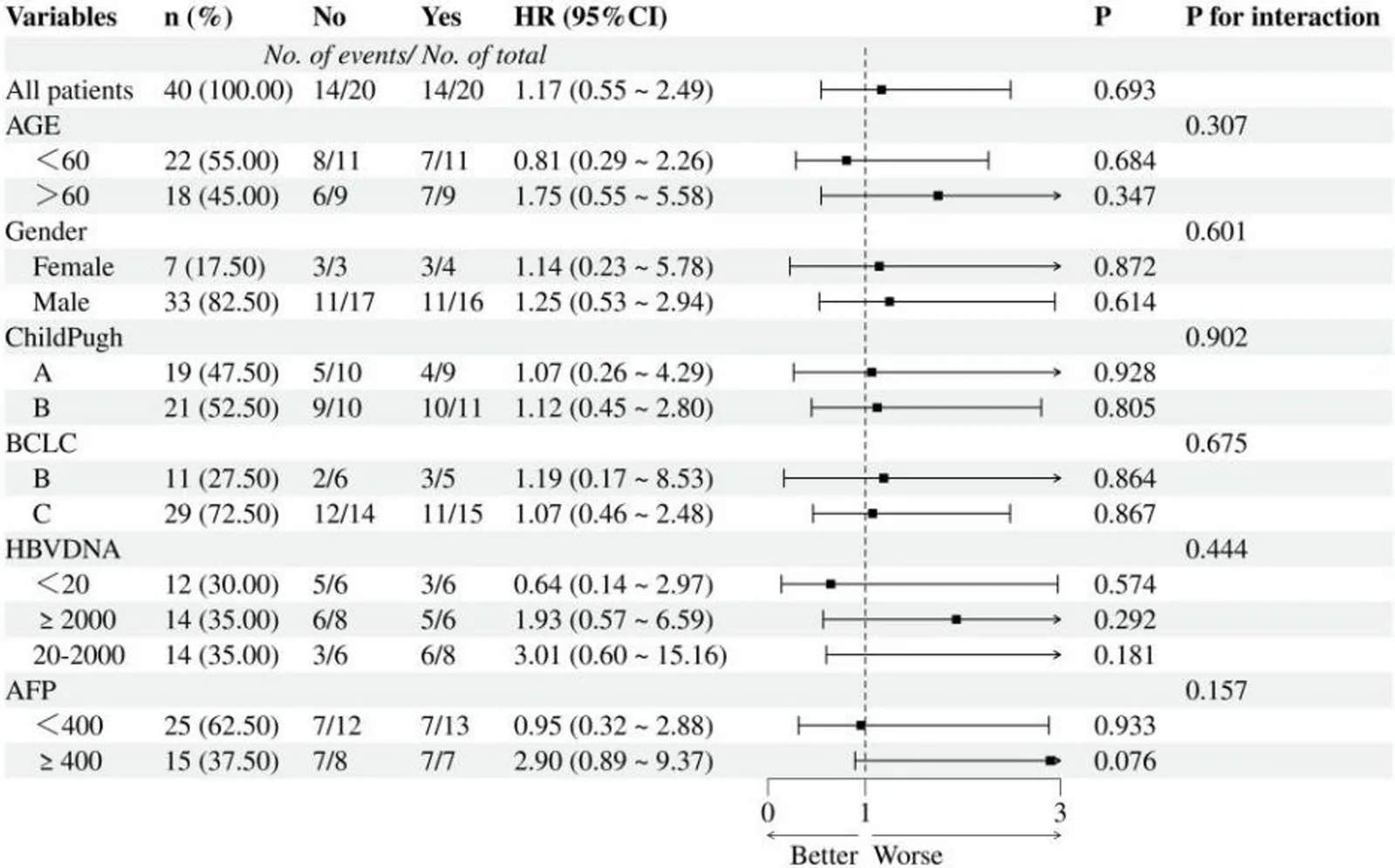
Subgroup analysis of OS for ATB use after PSM. HBVDNA(IU/ml), AFP(ng/ml)

**Fig. 4:**
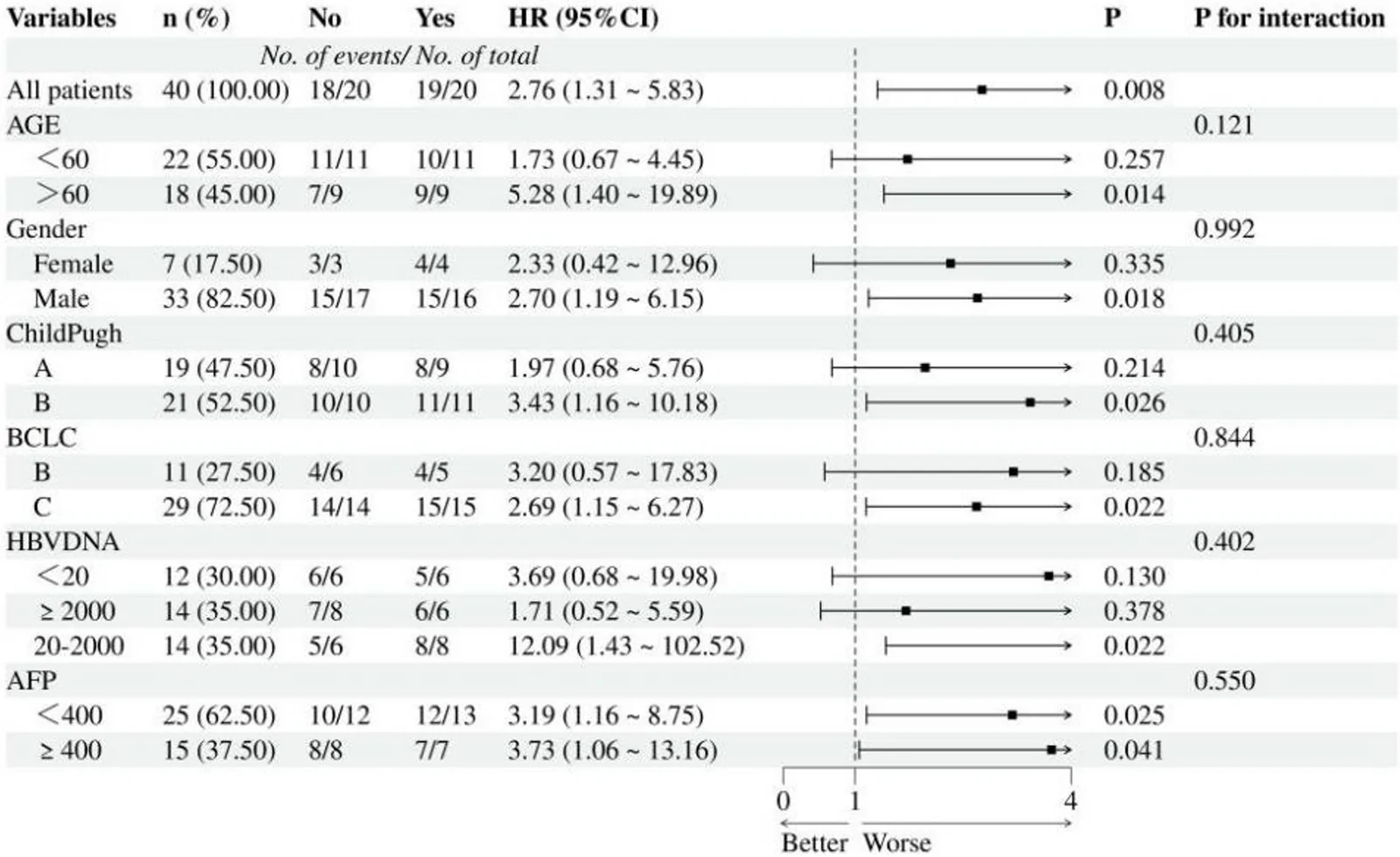
Subgroup analysis of PFS for ATB use after PSM. HBVDNA(IU/ml), AFP(ng/ml)

To explore whether the observed treatment effect varied by baseline characteristics, we pre-specified six clinically relevant subgroups before database lock: age (≤ 60 vs. > 60 years), gender (female vs. male), Child-Pugh class (A vs. B), BCLC stage (B vs. C), baseline HBV DNA (< 20, 20–2000, ≥ 2000 IU/mL) and AFP level (< 400 vs. ≥ 400 ng/mL). Subgroup definitions were chosen on the basis of their established prognostic value in HCC and their potential to modulate immunotherapy activity. Forest plots with Cox models stratified by subgroup were generated, and interaction terms (treatment × subgroup) were tested at a two-sided α = 0.10 level to flag potential effect modification. Across these subgroups, ATB exposure was not associated with improved OS (HR = 1.17; 95% CI 0.55–2.49; *P* = 0.693) but significantly increased the risk of progression (HR = 2.76; 95% CI 1.31–5.83; *P* = 0.008). Forest plots indicated that the deleterious effect was most prominent in patients aged > 60 years old, those with Child-Pugh class B, BCLC stage C, baseline HBV DNA: 20–2000 IU/mL or AFP ≥ 400 ng/mL, with corresponding HRs (95% CI) of 5.28 (1.40–19.89), 3.43 (1.16–10.18), 2.69 (1.15–6.27), 12.09 (1.43–102.52) and 3.73 (1.06–13.16), respectively. Formal interaction tests did not reach statistical significance (Figs. 3 and 4 ).

### Safety profile

Treatment-related adverse events were observed in 156 patients (69.6%). Before matching, the incidence of any-grade AEs was comparable between the ATB and non-ATB groups (60.0% vs. 70.9%; *P* = 0.266); similar findings were seen after matching (65.0% vs. 75.0%; *P* = 0.490). Rates of grade 3–4 toxicity were also similar (pre-matching 12.0% vs. 7.5%; *P* = 0.701; post-matching 15.0% vs. 15.0%; *P* = 1.00) (Table 5).

**Table 5:** Occurrence of Adverse Events

Adverse Effects	Before PSM		After PSM	
	Non-ATB Use 1-2G/3-4G	ATB Use 1-2G/3-4G	Non-ATB Use 1-2G/3-4G	ATB Use 1-2G/3-4G
Increased ALT/AST	34/12	3/2	4/3	3/2
Fever	9/0	2/0	1/0	2/0
Itching	4/0	0/0	1/0	0/0
Weakness	32/1	2/1	3/0	2/1
Decreased appetite	25/0	3/0	4/0	2/0
Abdominal distension	8/0	2/0	1/0	1/0
Diarrhea	7/1	2/0	1/0	2/0
Hypertension	6/0	0/0	0/0	0/0
Desquamate	8/0	0/0	0/0	0/0
Hypothyroidism	2/1	0/0	0/0	0/0
Hyperthyroidism	4/0	0/0	0/0	0/0
Myocarditis	2/0	1/0	0/0	1/0
Any-grade AE, *n*(%)	141 (70.9)	15 (60.0)	15 (75.0)	13 (65.0)
Grade 3–4 AE, *n*(%)	15 (7.5)	3 (12.0)	3 (15.0)	3 (15.0)

*Abbreviations*: *AE* adverse event, *PSM* propensity score matching, *ATB* antibiotics, *G* grade

*P*-value for any-grade AEs before PSM: 0.266; grade 3–4 AEs before PSM: 0.701

*P*-value for any-grade AEs after PSM: 0.490; grade 3–4 AEs after PSM: 1.00

## Discussion

Immune checkpoint inhibitors (ICIs) primarily reverse T-cell exhaustion, reshape antitumor immunity, and achieve durable cancer immune surveillance by disrupting the PD-1/PD-L1 axis to relieve immunosuppressive signaling within the tumor microenvironment (TME) [16–18]. However, a growing body of evidence suggests that concurrent use of baseline medications—especially antibiotics—attenuates the therapeutic efficacy of immune checkpoint inhibitors (ICIs) against cancer [19]. Experimental studies in mice reveal that antimicrobial exposure cultivates an immunosuppressive tumor microenvironment that facilitates immune escape [20]. The use of fluoroquinolones exhibits a sustained correlation with diminished clinical responses to immune checkpoint blockade [21, 22]. In extrahepatic solid tumours, Cortellini et al. demonstrated that antibiotic exposure significantly impaired outcomes after PD-1/PD-L1 blockade, increasing the hazard for both progression-free and overall survival [23]. Likewise, Elkrief et al. found that antibiotics conferred inferior prognosis in melanoma patients receiving ICIs [13].

A critical challenge in interpreting our findings is the inherent confounding between antibiotic exposure and infection severity. All patients in the ATB cohort received antibiotics for documented infections, raising the possibility that shortened PFS reflects immunosuppression induced by severe infection rather than antibiotic-mediated gut dysbiosis. Indeed, bloodstream and abdominal infections can trigger cytokine storms, lymphopenia, and acute-phase responses that independently compromise antitumor immunity. To address this, we performed a procalcitonin (PCT)-stratified survival analysis. PCT is a highly specific biomarker of bacterial infection severity; concentrations < 0.25 ng/mL are strongly associated with a decreased likelihood of detecting systemic bacterial infection and reduced disease severity, and this threshold is widely used in clinical practice to guide antibiotic stewardship decisions [14, 15]. Among the 85% of matched patients with PCT < 0.25 ng/mL, indicating minimal systemic infectious burden, ATB exposure remained significantly associated with shorter PFS (HR = 2.85, 95% CI 1.24–6.56; *P* = 0.014), with an effect size nearly identical to the overall cohort. This finding argues against an explanation based solely on infection-induced immunosuppression, as patients with low infectious burden still experienced substantially worse outcomes when exposed to antibiotics.

We propose a dual-pathway model: severe infection induces direct immunosuppression, while antibiotics independently impair immunotherapy efficacy through gut microbiota depletion. These pathways are not mutually exclusive and may be synergistic—patients with severe infections who receive broad-spectrum antibiotics face both insults simultaneously. Our data suggest that even in the absence of severe systemic infection (PCT < 0.25 ng/mL), antibiotic exposure alone is sufficient to compromise PFS, consistent with experimental evidence that antimicrobial-induced dysbiosis attenuates ICI efficacy by depleting commensal bacteria required for systemic immune priming [20, 24–26].

The therapeutic efficacy of the TACE-TKI-ICI triad in advanced HCC is predicated on a multi-level synergistic mechanism [3–5, 16–18]. First, TACE induces acute tumor ischemia and necrosis, leading to the release of tumor-associated antigens and damage-associated molecular patterns (DAMPs). This creates an immunogenic tumor microenvironment that enhances antigen presentation and primes adaptive immune responses [3, 4]. Second, tyrosine kinase inhibitors—particularly anti-angiogenic agents such as lenvatinib—normalize aberrant tumor vasculature by inhibiting VEGF signaling, thereby improving immune cell infiltration, reducing intratumoral hypoxia, and alleviating hypoxia-induced immunosuppression [3, 5]. Third, immune checkpoint inhibitors reverse T-cell exhaustion by disrupting the PD-1/PD-L1 axis, restoring cytotoxic T-cell function and sustaining anti-tumor immunity [16–18]. The convergence of these three modalities creates a favorable feedback loop: TACE-driven antigen release provides the “signal,” TKI-mediated vascular normalization facilitates immune cell access, and ICI unleashes the “brake” on T-cell activation.

Antibiotics may disrupt this carefully orchestrated synergy through gut microbiota-dependent and independent pathways [13, 19, 22]. The gut microbiome is integral to ICI efficacy; commensal bacteria such as Ruminococcaceae and Bacteroidetes species produce short-chain fatty acids (SCFAs) and other immunomodulatory metabolites that promote dendritic cell maturation, enhance antigen presentation in gut-associated lymphoid tissue, and facilitate systemic CD8 + T-cell priming [24–26]. Antibiotic-induced dysbiosis depletes these beneficial taxa, impairs intestinal barrier integrity, and reduces SCFA production, thereby attenuating the immunogenic effect of TACE and compromising ICI-driven T-cell activation [8, 10, 20]. Furthermore, antibiotics alter secondary bile acid metabolism through the gut-liver axis, creating a pro-inflammatory microenvironment that may accelerate HCC progression independently of direct anti-tumor effects [24, 25, 27]. These mechanisms collectively explain why antibiotic exposure in our cohort was associated with significantly shorter PFS despite comparable TACE intensity and ICI administration [28, 29].

In the present study, early antibiotic exposure did not significantly shorten overall survival (OS) in patients with primary hepatocellular carcinoma (HCC) receiving transcatheter arterial chemoembolization (TACE) plus immune checkpoint inhibitors (ICIs) and tyrosine kinase inhibitors (TKIs); however, it significantly shortened progression-free survival (PFS). This finding is consistent with several previous studies. Maesaka et al. reported that antibiotic pretreatment reduced disease control rate by 28%–30%, shortened median PFS by 6.1 months, and significantly decreased OS in 105 HCC patients treated with atezolizumab plus bevacizumab [30]. Similarly, Pinato et al. conducted a pooled analysis of 4,098 patients with advanced HCC and demonstrated that antibiotic use within 30 days before or after initiation of anticancer therapy was associated with significantly worse outcomes across various treatment modalities, including TKIs, ICI monotherapy, and combination regimens [12]. The underlying mechanism for these observations may involve antibiotic-induced gut dysbiosis, which can alter metabolite levels such as secondary bile acids through the gut-liver axis, thereby creating a pro-inflammatory and pro-tumorigenic microenvironment that accelerates chronic liver injury and HCC progression [24–28, 31].

However, our findings appear to conflict with those of Fessas et al. [11], who reported that early antibiotic exposure was associated with improved outcomes in HCC patients receiving immunotherapy. We hypothesize that this discrepancy may stem from differences in infection severity distribution. Fessas et al. included a substantial proportion of patients receiving prophylactic antibiotics without active infection, in whom the beneficial effects of preventing infection may have outweighed any microbiota-related harm. In contrast, our cohort comprised exclusively therapeutic antibiotic use for documented infections, where the dual insult of severe infection plus antibiotic-induced dysbiosis may have produced net harm. Notably, our PCT-stratified analysis demonstrated persistent harm even in patients with minimal infectious burden (PCT < 0.25 ng/mL), suggesting that antibiotic-induced microbiota perturbation may represent an independent contributor to immunotherapy failure. This interpretation underscores the critical importance of infection indication when evaluating antibiotic effects in oncology.

Further analysis in the present study, we identified antibiotic use, Child-Pugh class B, and HBV DNA ≥ 2,000 IU/mL as independent predictors of disease progression. Subgroup analyses indicated that this deleterious effect was most pronounced in patients aged > 60 years, those with Child-Pugh class B, BCLC stage C, baseline HBV DNA 20–2,000 IU/mL, or AFP ≥ 400 ng/mL, although formal interaction tests did not reach statistical significance. Multiple studies have demonstrated that antibiotics can significantly shorten median overall survival (OS) in patients receiving ICI therapy, and that antibiotic use serves as an independent risk factor for shortened progression-free survival (PFS), findings that align with our own results [32, 33]. Notably, Chen et al. performed a meta-analysis of prophylactic antibiotic use following TACE and found no significant reduction in infection-related parameters; conversely, prophylactic antibiotic administration significantly prolonged hospitalization duration, indicating limited benefit of routine prophylactic application [34].

Collectively, accumulating evidence consistently demonstrates that antibiotic exposure can impair antitumor immune responses through disruption of gut microbiota homeostasis and interference with glutamate/glutamine metabolism, thereby accelerating disease progression [29]. These findings underscore the necessity of implementing restrictive antibiotic stewardship strategies in high-risk HCC populations, with clinicians strictly adhering to prescribing indications and avoiding unnecessary antibiotic exposure to optimize immunotherapy outcomes.

The lack of standardized tumor response assessment in the original manuscript represented a limitation that we have now addressed. Retrospective application of mRECIST criteria revealed an ORR of 38.39% and DCR of 86.16% in the full cohort, consistent with published data for TACE + TKI+ICI combination therapy in advanced HCC. Notably, no patient achieved CR, which is attributable to the advanced disease stage (predominantly BCLC C), high prevalence of portal vein tumor thrombosis, and elevated baseline AFP in this real-world cohort. The consistent trend toward lower ORR and DCR in the ATB group—despite lacking statistical significance in the modest matched sample—corroborates the significantly shorter PFS observed in our primary analysis, supporting the hypothesis that antibiotic-induced gut dysbiosis impairs immunotherapy efficacy.

We excluded 17 patients who survived < 3 months from treatment initiation. While this decision was methodologically necessary to minimize immortal time bias, we acknowledge that early mortality may itself reflect treatment failure or antibiotic-related complications. Notably, among these excluded patients, the time to death was comparable between antibiotic-exposed and non-exposed groups (*P* = 0.291), and antibiotic use was predominantly observed in patients with Child-Pugh class B (*P* = 0.044), suggesting that hepatic decompensation rather than antibiotic exposure per se was the primary driver of early mortality. Nevertheless, this exclusion represents a potential selection bias that may limit generalizability.

Our investigation has limitations. Firstly, it is a single-centre, retrospective study with a modest matched sample. While the post-hoc power analysis indicated adequate power (approximately 89%) to detect the observed effect size (HR = 2.76) for the primary endpoint of PFS, the study was not powered a priori and may be underpowered for smaller effect sizes (HR < 2.5), to evaluate OS differences (which was non-significant), or to perform robust subgroup analyses by individual ICI agents or infection types. Treatment intensity: a post-baseline mediator rather than confounder, TACE sessions and ICI cycles were not included in the propensity-score matching model, as these are post-baseline variables. Post-hoc comparisons confirmed comparable TACE intensity between matched groups (*P* = 0.880). While the median ICI cycle count was identical (12 cycles), the broader distribution in the ATB group [IQR 4–12 vs. 11.25–12] likely reflects earlier discontinuation due to disease progression or infection, which is consistent with our primary finding of shorter PFS. This pattern supports our interpretation that differential ICI exposure was a mediator (consequence of antibiotic-induced treatment failure) rather than a confounder (independent determinant of outcome). TACE is an operator-dependent procedure; however, all procedures were performed by a consistent team of experienced interventional radiologists using a standardized institutional protocol, minimizing inter-operator variability. Secondly, despite rigorous propensity-score matching, residual confounding cannot be wholly eliminated. Large-scale, multicentre, prospective trials are needed to confirm the present findings, clarify the microbiological underpinnings and furnish high-level evidence for precision oncology in hepatocellular carcinoma. We were unable to perform subgroup analyses stratified by individual ICI agent due to the limited matched sample size (*n* = 40) and the identical distribution of ICI types after matching (*P* = 1.000; Supplementary Table S3). While the three anti-PD-1 antibodies share the same target, subtle differences in binding affinities and Fc region engineering may result in differential interactions with the gut microbiome and antibiotic-induced dysbiosis. Future studies with larger cohorts are needed to investigate whether specific ICI agents are more or less susceptible to antibiotic-mediated efficacy reduction.

Thirdly, confounding by infection severity. The most important limitation of our study is the inability to fully decouple the immunosuppressive effects of severe infection from the microbiota-disrupting effects of antibiotics. All ATB-exposed patients had documented infections, and no ‘infected without antibiotics’ control group was available, reflecting ethical standards of care. Although PCT-stratified analysis suggested persistent harm in patients with minimal infectious burden (PCT < 0.25 ng/mL)—a threshold established in clinical guidelines to exclude systemic bacterial infection [14, 15] —we cannot exclude residual confounding from unmeasured infection-related factors (e.g., duration of bacteremia, septic shock, ICU admission, or unquantified viral/fungal co-infections). The PCT ≥ 0.25 ng/mL subgroup was too small (*n* = 6) for reliable effect estimation. Future prospective studies should incorporate comprehensive infection severity scores (SOFA, qSOFA), microbiological cultures, and gut microbiome sequencing to clarify the relative contributions of infection and antibiotics to immunotherapy failure.

## Conclusion

Antibiotic exposure significantly impairs progression-free survival (PFS) in patients with HBV-related intermediate-to-advanced hepatocellular carcinoma undergoing TACE plus ICIs and TKIs, representing an independent prognostic factor. Unnecessary antibiotic use should be avoided, particularly in subgroups aged > 60 years, with Child-Pugh class B, BCLC stage C, baseline HBV DNA 20–2000 IU/mL, or AFP ≥ 400 ng/mL. Notably, antibiotic administration did not affect adverse event incidence in this cohort.

## Supplementary Information

Supplementary Material 1.

## Data Availability

The datasets used and analysed during the current study are available from the corresponding author on reasonable request.

## Abbreviations

ATB: Antibacterials
ALB: Albumin
ALT: Alanine Aminotransferase
AST: Aspartate aminotransferase
HCC: Hepatocellular Carcinoma
HBV: Hepatitis B virus
ICI: Immune checkpoint inhibitors
LYM: Lymphocyte
NE: Neutrophil
PLT: Platelet
PSM: Propensity-score Matching
TKI: Tyrosine kinase inhibitor
TACE: Transcatheter arterial chemoembolization
TBIL: Total bilirubin
WBC: White blood cell
