# Supplementary material for: Early antibiotic exposure reduces progression-free survival in unresectable hepatocellular carcinoma patients treated with TACE plus TKI and ICI therapy: a propensity score-matched analysis
Source: BMC Gastroenterol. 2026 Jul 15;26:622. doi: 10.1186/s12876-026-05126-4 (PMC13647644; doi:10.1186/s12876-026-05126-4)
Supplement: Supplementary file 1 [file 12876_2026_5126_MOESM1_ESM.docx]

| **Supplementary Table S1. Baseline Characteristics of Excluded Patients** | | | | |
| --- | --- | --- | --- | --- |
| **Variables** | **Total (n = 17)** | **Non-ATB(n = 12)** | **ATB(n = 5)** | **P** |
| AGE, years | 59.00 (53.00, 63.00) | 57.50 (53.75, 60.25) | 67.00 (53.00, 68.00) | 0.46 |
| Survavil,days | 68.00 (51.00, 81.00) | 62.00 (48.00, 73.50) | 68.00 (59.00, 88.00) | 0.291 |
| HBVDNA, n(%) |  |  |  | 0.406 |
| <20 IU/mL | 5 (29.41) | 2 (16.67) | 3 (60.00) |  |
| ≥2000 IU/mL | 7 (41.18) | 6 (50.00) | 1 (20.00) |  |
| 20-2000 IU/mL | 5 (29.41) | 4 (33.33) | 1 (20.00) |  |
| AFP, n(%) |  |  |  | 0.62 |
| <400 ng/mg | 9 (52.94) | 7 (58.33) | 2 (40.00) |  |
| ≥400 ng/mg | 8 (47.06) | 5 (41.67) | 3 (60.00) |  |
| Child-Pugh, n(%) |  |  |  | 0.044 |
| A | 10 (58.82) | 5 (41.67) | 5 (100.00) |  |
| B | 7 (41.18) | 7 (58.33) | 0 (0.00) |  |
| BLCL, n(%) |  |  |  | 1 |
| B | 3 (17.65) | 2 (16.67) | 1 (20.00) |  |
| C | 14 (82.35) | 10 (83.33) | 4 (80.00) |  |
| Abbreviations: ATB, antibiotics; M, median; Q₁, first quartile; Q₃, third quartile; HBV DNA, hepatitis B virus DNA; AFP, alpha-fetoprotein; NLR, neutrophil-to-lymphocyte ratio; BCLC, Barcelona Clinic Liver Cancer; AntiHBV, anti-hepatitis B virus therapy. | | | | |

| **Supplementary Table S2. mRECIST response assessment in patients with HBV-related advanced hepatocellular carcinoma treated with TACE plus TKI and ICI, before and after propensity-score matching.** | | | | | | | | |
| --- | --- | --- | --- | --- | --- | --- | --- | --- |
| **Variables** | **Before PSM** | | | | **After PSM** | | | |
|  | **Total(n = 224)** | **Non-ATB(n = 199)** | **ATB (n = 25)** | **P** | **Total (n = 40)** | **Non-ATB(n = 20)** | **ATB(n = 20)** | **P** |
| **mRECIST, n(%)** |  |  |  | **0.289** |  |  |  | **0.327** |
| CR | 0(0) | 0(0) | 0(0) |  | 0(0) | 0(0) | 0(0) |  |
| SD | 107 (47.77) | 96 (48.24) | 11 (44.00) |  | 19 (47.50) | 8 (40.00) | 11 (55.00) |  |
| PR | 86 (38.39) | 78 (39.20) | 8 (32.00) |  | 15 (37.50) | 10 (50.00) | 5 (25.00) |  |
| PD | 31 (13.84) | 25 (12.56) | 6 (24.00) |  | 6 (15.00) | 2 (10.00) | 4 (20.00) |  |
| **ORR** | **86 (38.39)** | **78 (39.20)** | **8 (32.00)** |  | **15 (37.50)** | **10 (50.00)** | **5 (25.00)** |  |
| **DCR** | **193 (86.16)** | **174 (87.44)** | **19 (76.00)** |  | **34 (85.00)** | **18 (90.00)** | **16 (80.00)** |  |
| Abbreviations: mRECIST, modified Response Evaluation Criteria in Solid Tumors; ORR, objective response rate; DCR, disease control rate; CR, complete response; PR, partial response; SD, stable disease; PD, progressive disease. Note: ORR = CR + PR; DCR = CR + PR + SD. P-value calculated by Fisher's exact test. | | | | | | | | |

| **Supplementary Table S3. Distribution of immune checkpoint inhibitor types in patients with HBV-related advanced hepatocellular carcinoma treated with TACE plus TKI and ICI, before and after propensity-score matching.** | | | | | | | | |
| --- | --- | --- | --- | --- | --- | --- | --- | --- |
| **Variables** | **Before PSM** | | | | **After PSM** | | | |
|  | **Total(n = 224)** | **No(n = 199)** | **Yes (n = 25)** | **P** | **Total (n = 40)** | **Non-ATB(n = 20)** | **ATB(n = 20)** | **P** |
| **ICI types, n(%)** |  |  |  | **0.519** |  |  |  | 1.000 |
| camrelizumab | 155 (69.20) | 135 (67.84) | 20 (80.00) |  | 33 (82.50) | 16 (80.00) | 17 (85.00) |  |
| sintilimab | 34 (15.18) | 31 (15.58) | 3 (12.00) |  | 5 (12.50) | 3 (15.00) | 2 (10.00) |  |
| tislelizumab | 35 (15.62) | 33 (16.58) | 2 (8.00) |  | 2 (5.00) | 1 (5.00) | 1 (5.00) |  |
| Abbreviations: ICI, immune checkpoint inhibitor; PSM, propensity score matching; ATB, antibiotics; n, number. | | | | | | | | |
| Note: All three agents are anti-PD-1 monoclonal antibodies administered at a fixed dose of 200 mg every 3–4 weeks. Camrelizumab was manufactured by Hengrui Medicine (Jiangsu, China), sintilimab by Innovent Biologics (China), and tislelizumab by BeiGene (Beijing, China). P-value calculated by Fisher's exact test. | | | | | | | | |

| **Supplementary Table S4. Subgroup analysis of progression-free survival by baseline procalcitonin (PCT) level after propensity-score matching** | | | | | | |
| --- | --- | --- | --- | --- | --- | --- |
| **Variables** | **n (%)** | **No** | **Yes** | **HR (95%CI)** | **P** | **P for interaction** |
| **All patients** | 40 (100.00) | 18/20 | 19/20 | 2.76 (1.31 ~ 5.83) | 0.008 |  |
| **PCT** |  |  |  |  |  | 0.647 |
| ＜0.25 ng/mL | 34 (85.00) | 14/15 | 18/19 | 2.85 (1.24 ~ 6.56) | 0.014 |  |
| ≥0.25 ng/mL | 6 (15.00) | 4/5 | 1/1 | 3655519970.88 (0.00 ~ Inf) | 0.999 |  |
| Abbreviations: PCT, procalcitonin; HR, hazard ratio; CI, confidence interval; ATB, antibiotics; NE, not estimable due to insufficient events. | | | | | | |
| Note: The PCT ≥0.25 ng/mL subgroup comprised only 6 patients (5 non-ATB vs 1 ATB with progression events), yielding unstable estimates. PCT <0.25 ng/mL indicates minimal systemic bacterial infectious burden according to established clinical thresholds | | | | | | |
